# Characterization and identification of protein O-GlcNAcylation sites with substrate specificity

**DOI:** 10.1186/1471-2105-15-S16-S1

**Published:** 2014-12-08

**Authors:** Hsin-Yi Wu, Cheng-Tsung Lu, Hui-Ju Kao, Yi-Ju Chen, Yu-Ju Chen, Tzong-Yi Lee

**Affiliations:** 1 Institute of Chemistry, Academia Sinica, Taipei 115, Taiwan; 2 Department of Computer Science and Engineering, Yuan Ze University, Taoyuan 320, Taiwan; 3 Innovation Center for Big Data and Digital Convergence, Yuan Ze University, Taoyuan 320, Taiwan

**Keywords:** O-GlcNAcylation, O-linked glycosylation, substrate site specificity, support vector machine

## Abstract

**Background:**

Protein O-GlcNAcylation, involving the attachment of single *N*-acetylglucosamine (GlcNAc) to the hydroxyl group of serine or threonine residues. Elucidation of O-GlcNAcylation sites on proteins is required in order to decipher its crucial roles in regulating cellular processes and aid in drug design. With an increasing number of O-GlcNAcylation sites identified by mass spectrometry (MS)-based proteomics, several methods have been proposed for the computational identification of O-GlcNAcylation sites. However, no development that focuses on the investigation of O-GlcNAcylated substrate motifs has existed. Thus, we were motivated to design a new method for the identification of protein O-GlcNAcylation sites with the consideration of substrate site specificity.

**Results:**

In this study, 375 experimentally verified O-GlcNAcylation sites were collected from dbOGAP, which is an integrated resource for protein O-GlcNAcylation. Due to the difficulty in characterizing the substrate motifs by conventional sequence logo analysis, a recursively statistical method has been applied to obtain significant conserved motifs. To construct the predictive models learned from the identified substrate motifs, we adopted Support Vector Machines (SVMs). A five-fold cross validation was used to evaluate the predictive model, achieving sensitivity, specificity, and accuracy of 0.76, 0.80, and 0.78, respectively. Additionally, an independent testing set, which was really blind to the training data of predictive model, was used to demonstrate that the proposed method could provide a promising accuracy (0.94) and outperform three other O-GlcNAcylation site prediction tools.

**Conclusion:**

This work proposed a computational method to identify informative substrate motifs for O-GlcNAcylation sites. The evaluation of cross validation and independent testing indicated that the identified motifs were effective in the identification of O-GlcNAcylation sites. A case study demonstrated that the proposed method could be a feasible means of conducting preliminary analyses of protein O-GlcNAcylation. We also anticipated that the revealed substrate motif may facilitate the study of extensive crosstalk between O-GlcNAcylation and phosphorylation. This method may help unravel their mechanisms and roles in signaling, transcription, chronic disease, and cancer.

## Introduction

Protein O-GlcNAcylation is an O-linked glycosylation involving the β-attachment of a single N-acetylglucosamine (GlcNAc) to the serine (Ser)/threonine (Thr) residues, adding 203.07 Da to the modified proteins [1]. Two enzymes, O-GlcNAc transferase (OGT) and O-GlcNAcase (OGA), are responsible for the addition and removal of O-GlcNAc, respectively. O-GlcNAc has been found on a myriad of cytoplasmic and nuclear proteins and has the ability to modulate molecular processes such as transcription, translation, protein stability, and signal transduction, as well as cellular processes including proliferation, apoptosis and development [2]. Disregulation of O-GlcNAcylation has been found in diseases such as diabetes [3] and Alzheimer disease [4]. O-GlcNAcylation modifies proteins at serine/threonine residues and thus has been proposed to potentially play a role in modulating protein function by affecting protein phosphorylation [5].

Due to its labile, dynamic, and substoichiometric characteristics, the precise identification of O-GlcNAcylation sites by advanced systematic proteomic approaches remains challenging [6]. Liquid chromatography mass spectrometry (LC-MS/MS)-based techniques are most utilized for detection and site specific identification of O-GlcNAcylation. As the improvement of mass spectrometry technologies and the enrichment methods, many O-GlcNAcylated proteins in postsynaptic density [7], murine synapse [8], mouse brain [9], rat brain [10], mouse embryonic stem cell [11], and hela cells [12], were identified in recent years. The system-wide interplay between O-GlcNAcylation and phosphorylation were also studied [13,14]. Due to the growing interest in revealing the O-GlcNAcylation site and attempting to reduce experimental efforts, computational prediction of O-GlcNAcylation sites and conserved motifs becomes important. In 2002, Gupta and Brunak have developed a prediction program termed YinOYang that was trained with 40 O-GlcNAccylation sites [15]. In 2011, Wang et al. have developed OGlcNAcScan that was trained with 373 O-GlcNAcylation sites [16]. In 2013, O-GlcNAcPRED has been proposed and claimed to have better performance than these two aforementioned predictors [17].

Although several methods have been proposed for the computational identification of O-GlcNAcylation sites, so far, no tools focused on the investigation of O-GlcNAcylated substrate motifs. Thus, we were motivated to characterize the O-GlcNAcylation sites with the consideration of substrate specificity. Here, we intended to predict O-GlcNAcylated sites along with their potential substrate motifs by using a statistical method. The substrate motifs were further analyzed for the interplay between phosphorylation and O-GlcNAcylation. To facilitate the study of protein O-GlcNAcylation, the identified substrate motifs could be exploited to implement a prediction tool for identifying O-GlcNAcylation sites with potential substrate motifs.

## Materials and methods

### Data collection and preprocessing

The experimentally verified O-GlcNAcylation sites were mainly extracted from dbPTM [18-20] which have integrated several protein glycosylation-associated databases: dbOGAP [16], UniProtKB [21], O-GlycBase [22], and PhosphoSitePlus [23]. The dbOGAP database contains 240 and 135 sites for O-GlcNAcylated serine (Ser) and threonine (Thr) on 168 proteins. O-GlcNAcylation data, from UniProtKB, that are experimentally verified and annotated as "by similarity", "potential", "probable" were removed, resulting in 57 and 51 sites for O-GlcNAcylated Ser and Thr on 51 proteins. In O-GlycBase version 6.0, there are 24 sites for O-GlcNAcylated Ser and Thr from 17 proteins. In particular, the protein phosphorylation database, PhosphoSitePlus, also manually curated the experimental data of other PTM types such as acetylation, glycosylation, ubiquitylation, sumoylation, and so on. Totally 779 and 582 experimentally verified sites for O-GlcNAcylated Ser and Thr on 542 proteins were obtained from PhosphoSitePlus.

In this work, the experimental data of 375 O-GlcNAcylation sites from dbOGAP was defined as the positive training data. Referring to KinasePhos [24,25], a window of 11 amino acids with O-GlcNAcylated Ser or Thr residues at the center was used to investigate the surrounding residues. The same sequence window size centered on non-O-GlcNAcylated Ser and Thr residues were used as negative training data. A total of 16740 and 10079 negative sequence fragments for Ser and Thr residues were obtained on 168 proteins from dbOGAP (Table 1). Balancing the negative and positive training data, a biased prediction performance for a binary classification between positive and negative data was avoided. Among previous methods predicting phosphorylation [26-31], a *K*-means clustering method [32,33] was used to generate a negative data set. The value of *K *representing the number of subsequent positive data, indicated the number of samples obtained from the negative set. As shown in Table 1 positive and negative sequence fragments were in the training data.

**Table 1:** Number of sites of training and independent testing set.

Data resource			O-GlcNAcylated sites (Positive data)	Non-O-GlcNAcylated sites (Negative data)
**Training set**	dbOGAP	Serine	240	16740
		
		Threonine	135	10079
		
		Ser and Thr	375	26819

**Independent testing set**	UniProtKB	Serine	57	4488
		
		Threonine	51	2978
		
		Ser and Thr	108	7466
	
	OGlycBase	Serine	24	1013
		
		Threonine	24	694
		
		Ser and Thr	48	1707
	
	PhosphoSitePlus	Serine	779	58082
		
		Threonine	582	34217
		
		Ser and Thr	1361	92299
	
	**Non-redundant dataset**	Serine	578	41075
		
		Threonine	470	23920
		
		Ser and Thr	1048	64995

In the prediction of O-GlcNAcylation sites, the performance of the predictive models may be overestimated owing to the over-fitting of a training set. To estimate the real predictive performance, the experimental data obtained from UniProtKB, O-GlycBase, and PhosphoSitePlus was considered as the independent testing set. Data from one database was compared to that from the other databases using their O-GlcNAc modified position and the UniProtKB accession number. Overlapped data set was removed to prevent redundancy. After the removal of redundant data, we have obtained 578 and 470 positive sequence fragments as well as 41075 and 23920 negative sequence fragments of Ser and Thr residues for independent testing.

### Detection of O-GlcNAcylated site specificities

**Figure 1:**
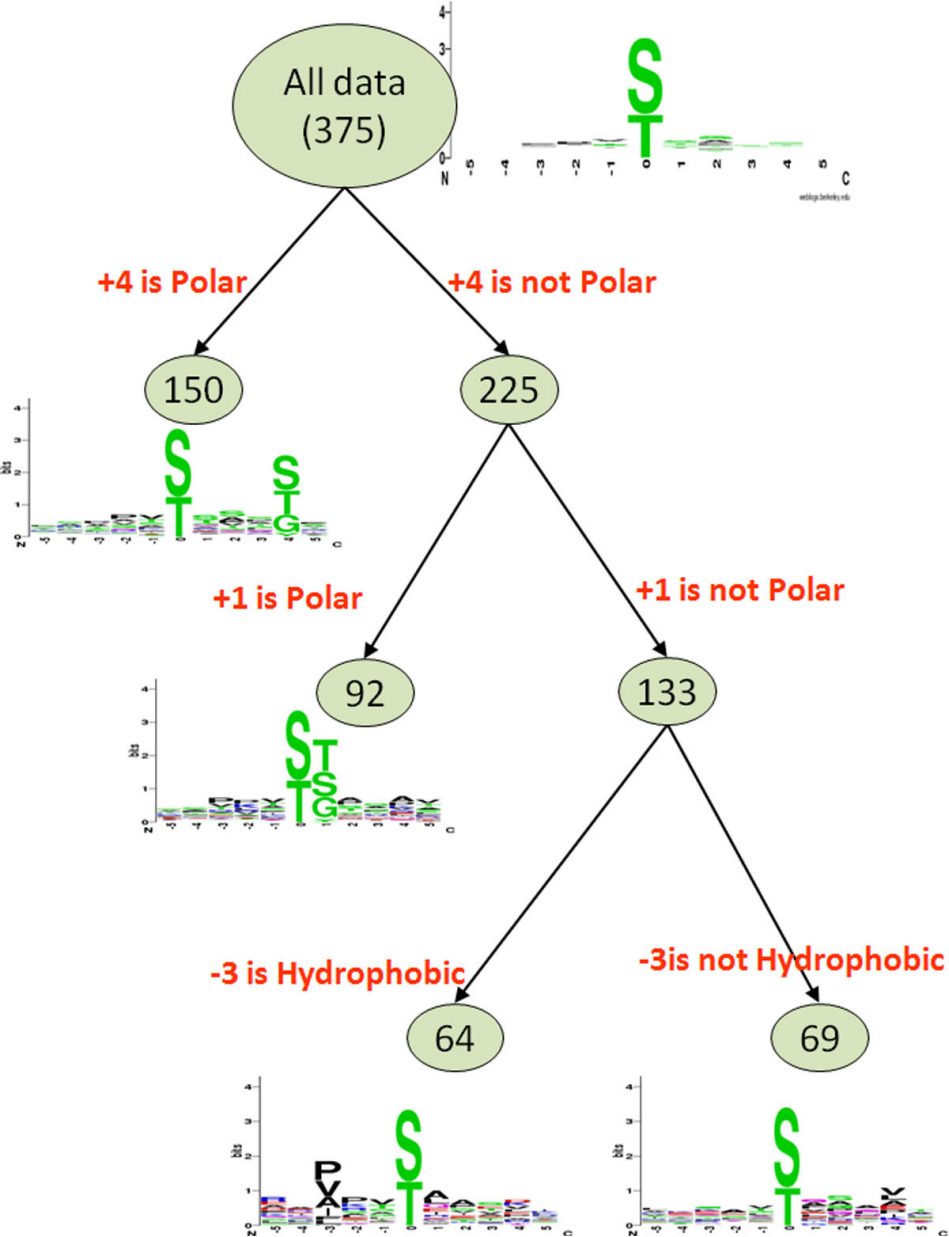
**The tree-like visualization of the identified substrate motifs by applying MDDLogo**.

In order to obtain substrate motif signatures of O-GlcNAcylation sites, the positive training data was analyzed by a motif analysis tool, MDDLogo [33]. The MDDLogo clustered a set of aligned O-GlcNAcylated sequences to divide a large group into subgroups that contain statistically significant substrate motifs. It has been suggested that the grouping of protein sequences into smaller groups is prior to computationally identifying PTM sites [24-26,29,34-38]. To calculate the frequency of amino acid occurrence between two positions, *A_i _*and *A_j_*, that were proximal to the O-GlcNAcylated site, MDDLogo using chi-square test was applied. As listed in Supplementary Table S1 (Additional File 1), twenty amino acids were categorized into five groups according to their chemical properties (acidic, polar, basic, aromatic, and hydrophobic groups) to facilitate extracting motifs presenting conserved biochemical properties. Then, a contingency table of the amino acids occurrence between two positions was constructed. The chi-square test was defined as:

(1) $$\chi^{2}\left({\text{A}}_{\text{i}}\text{,}{\text{A}}_{\text{j}}\right)=\overset{5}{\underset{m=1}{\sum }}\overset{5}{\underset{n=1}{\sum }}\frac{{\left(X_{mn}-E_{mn}\right)}^{2}}{E_{mn}}$$

where *X_mn _*represented the number of sequences that had the amino acids of group *m *in position *A_i _*and had the amino acids of group *n *in position *A_j_*, for each pair (*A_i _*, *A_j_*) with *i*≠j. *E_mn _*was calculated as $\frac{X_{mR}\cdot X_{Cn}}{X}$, where *X_mR _*(the total number of sequences) = *X_m1_*+ ... +*X_m5_*, *X_Cn _*= *X_1n_*+ ... +*X_5n_*, and *X*. Once *X^2 ^*was estimated larger than 34.3, which suggests *p*<0.005 with 16 degrees of freedom between two positions and considered as strong dependence, the process was carried on as described in a previous work [39]. Figure 1 shows an example of this process. Maximal dependence occurred on position +4 which represents the occurrence of polar group. According to that, two subgroups were generated illustrating the occurrence and absence of polar amino acids on position +4. The positive data was divided into tree-like subgroups based on a recursive clustering process. While applying MDDLogo method to the positive training data, the minimum cluster size needed to be determined to cluster the sequences fragments. The clustering of the subgroup will be suspended once the data size of a subgroup was less than the user-determined minimum cluster size. An optimal minimum cluster size can be yielded from performing MDDLogo using various values. Subgroup derived from MDDLogo was depicted by using WebLogo [40] which help verify whether conserved motifs of O-GlcNAc modified sites were existed or not.

### Features extraction and encoding

Aside from the component of flanking amino acids (AA), the evolutionary information and accessible surface area (ASA) surrounded the O-GlcNAcylation sites were also studied. Fragments of amino acids were extracted from positive and negative training sets using a window of length 2*n*+1 centered on substrate sites, where *n *was set to five in this study. An orthogonal binary coding scheme was adopted to transform amino acids into numeric vectors, in the so-called 20-dimensional binary coding. For example, glycine was encoded as "10000000000000000000;" alanine was encoded as "01000000000000000000," and so on. The number of feature vectors that represented the flanking amino acids surrounding the O-GlcNAcylation site was (2*n*+1) × 20. A total of *p *vectors {*x_i_*, *i *= 1, ..., *p*} were used, to represent all *p *sequence fragments in the training data. Each vector was labeled with the class of its corresponding protein (e.g. positive or negative). For the composition of 20 amino acids surrounding the O-GlcNAcylation sites, the vector xi included 20 elements for the amino acid composition (AAC) and 400 elements for the amino acid pair composition (AAPC). The 20 elements specified the numbers of occurrences of 20 amino acids normalized with the total number of residues in a sequence fragment, and the 400 elements specified the numbers of occurrences of 400 amino acid pairs normalized with the total number of residue pairs in a sequence fragment.

To determine the positional weighted matrix (PWM) of amino acids close to the O-GlcNAcylated sites, non-homologous training data and SulfoSite method [41] was used. The relative frequency of amino acids that surrounded the O-GlcNAc sites and fragment sequence were denoted and encoded by PWM, respectively. A matrix, containing (2*n*+1) × *w *elements, profiled the distribution of amino acids of the training dataset. Here, 2*n*+1 denoted the window size while *w *was composed of 20 amino acids and 1 terminal signal.

From the viewpoint of structural environment, several amino acid residues of a protein can be mutated without changing its structure, and two proteins may have similar structures with different amino acid compositions. Position Specific Scoring Matrix (PSSM) profiles, which have been extensively utilized in protein secondary structure prediction, subcellular localization and other bioinformatics problems [42-45], were adopted herein with significant improvement. The PSSM profiles were obtained by PSI-BLAST [46] against non-redundant sequences of O-GlcNAcylated sites. Supplementary Figure S1 (Additional File 1) displayed in detail how to generate the 400D PSSM features for each sequence fragment. The matrix of (2*n*+1) × 20 elements had rows centered on substrate site, extracted from the PSSM profile, where 2*n*+1 represented the window size and 20 represents the position specific scores for each type of amino acid. Then, the (2*n*+1) × 20 matrix was transformed into a 20 × 20 matrix by summing up the rows that were associated with the same type of amino acid. Finally, every element in 20 × 20 matrix was divided by the window length 2*n*+1 and then was normalized using the formula: $\frac{1}{1+e^{-x}}$.

It has been reported by Pang *et al*. [47] that proteins that had post-translational modifications made the modified amino acids more accessible on the protein surface. To investigate if this character can be used to discriminate the O-GlcNAc modified sites from other residues, the solvent-accessible surface area (ASA) was employed. Due to the lack of protein tertiary structures for most O-GlcNAcylated proteins in PDB [48], with reference to a previous method [32], an effective tool, RVP-Net [49,50], was applied to compute the ASA value from protein sequence, showing the proportion of the solvent-accessible area for each amino acid on proteins. Briefly, ASA value of all residues came from RVP-Net utilizing full-length protein sequences annotated with experimentally verified O-GlcNAcylation sites as input data. Finally, the ASA values were normalized to be 0~1 for every amino acids close to the O-GlcNAcylation sites.

### Model construction and cross-validation

In this work, the predictive model was learned from the data of the training set by Support Vector Machine (SVM) whose concept was based on binary classification. The kernel function then projected the input samples into a higher dimensional space to locate a hyper-plane that can distinguish the two classes with maximal margin and minimal error. Predictive models that has been trained with various features were obtained by using LIBSVM [51], a public SVM library. The kernel function of the SVMs was the radial basis function (RBF), defined as $K\left(S_{i},S_{j}\right)=\text{exp}\left(-\gamma {∥S_{i}-S_{j}∥}^{2}\right)$. LIBSVM library yielded a value of probability ranging from 0 to 1 for each prediction, among which, the probability came from the classifier trained with the best feature were used as an input vector for second-layered SVM.

To construct a final model, five-fold cross validation was performed to evaluate the predictive performance of each model using different features. In order to achieve this, each dataset was divided into five approximately equal sized subgroups, of which, 1 and other 4 subgroups functioned as the test and training dataset, respectively, during cross-validation. Cross-validation process was performed five times so that each subgroup can be used as the test set. The five validation results were then combined to produce a single result. The advantage of cross-validation evaluation was that all original data was tested only once, but distributed into the test and training sets [52]. Here, we adopted four measures to evaluate the predictive performance of the trained models: Sensitivity (Sn) = TP/(TP+FN), Specificity (Sp) = TN/(TN+FP), Accuracy = (TP + TN)/(TP+FP+TN+FN) [53], and Matthews Correlation $\text{Coefficient }\left(\text{MCC}\right)=\frac{\left(TP\times TN\right)-\left(FN\times FP\right)}{\sqrt{\left(TP+FN\right)\times \left(TN+FP\right)\times \left(TP+FP\right)\times \left(TN+FN\right)}}$, where TP, TN, FP and FN represent the numbers of true positives, true negatives, false positives, and false negatives, respectively. Once the selection of the predictive model with best performance has been accomplished, the predictive performance of the best model was further estimated by an independent testing.

## Results and discussion

### Substrate site investigation

**Figure 2:**
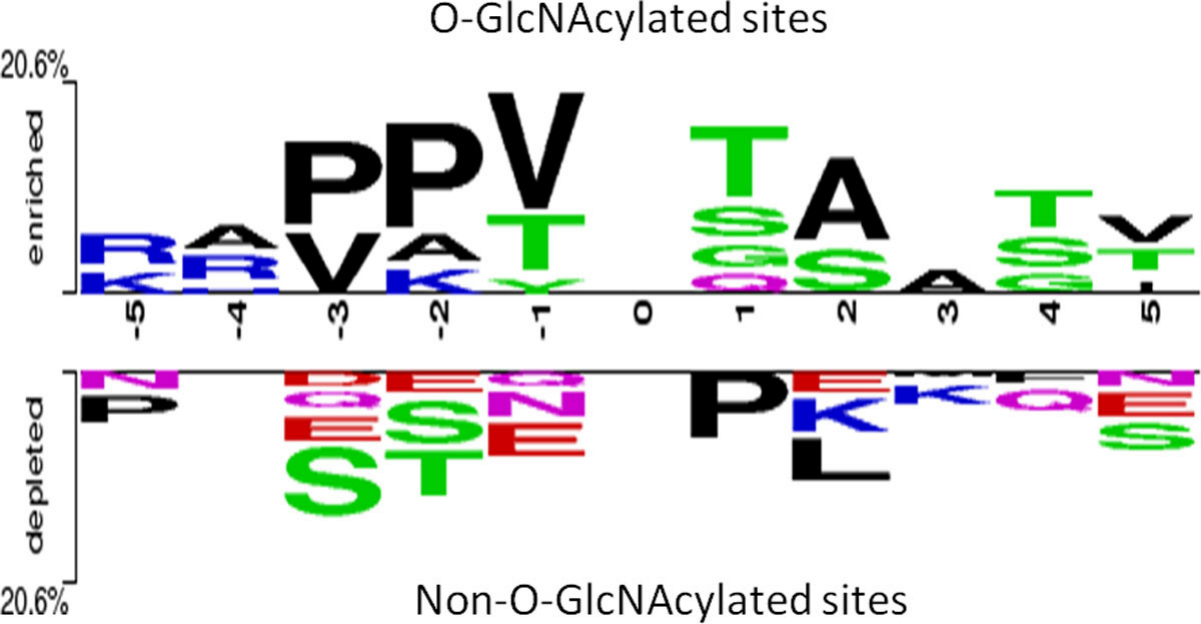
**TwoSampleLogo between O-GlcNAcylated and non-O-GlcNAcylated sites**.

This study intended to study substrate specificity for O-GlcNAcylation based on the sequence-based analysis. In order to explore the amino acid composition neighboring the O-GlcNAcylated Ser/Thr residues, TwoSampleLogo, a web-based tool using multiple sequence alignments, that detected and displayed significant differences for compositions of sequence between two sets, was applied [54]. O-GlcNAcylated Ser/Thr (positive data) residues and unmodified ones (negative data) were centered on position 0, and the neighboring residues (-5~+5) were visualized by graphical sequence logos. Figure 2 presented the specific difference of amino acid positions and compositions between O-GlcNAcylation sites (375 sequences) and non-O-GlcNAcylation sites (26819 sequences). We observed that the most distinct feature of O-GlcNAcylation sites was hydrophobic amino acids, Proline (P), Valine (V), and Alanine (A), locating centrally around position -2 and +3. Supplementary Figure S2 (Additional File 1) indicated that the O-GlcNAcylation sites had a lower percentage of solvent-accessible surface area than non-O-GlcNAcylation sites, which was feasible to the abundance of hydrophobic amino acids surrounding substrate sites. Besides, the polar amino acids, Threonine (T) and Serine (S), also located centrally at position -1 and +1. Additionally, the positively charged Lysine (K) and Arginine (R) were dominant at position -2, -4 and -5, suggesting that the distant amino acids in sequence, which may be close to O-GlcNAcylation sites in three-dimensional structure, showed prominent difference between modified and unmodified sites. Another characteristic was the depletion of P and L at +1 and +2, respectively which was immediately adjacent to the O-GlcNAcylation sites. Absence of S, T, and Glutamate (E) were also found around position -2, -3, and +5. The overall motif extracted in this study is consistent with that consensus sequence previously suggested as P-P-T-[ST]-T-A [16].

**Figure 3:**
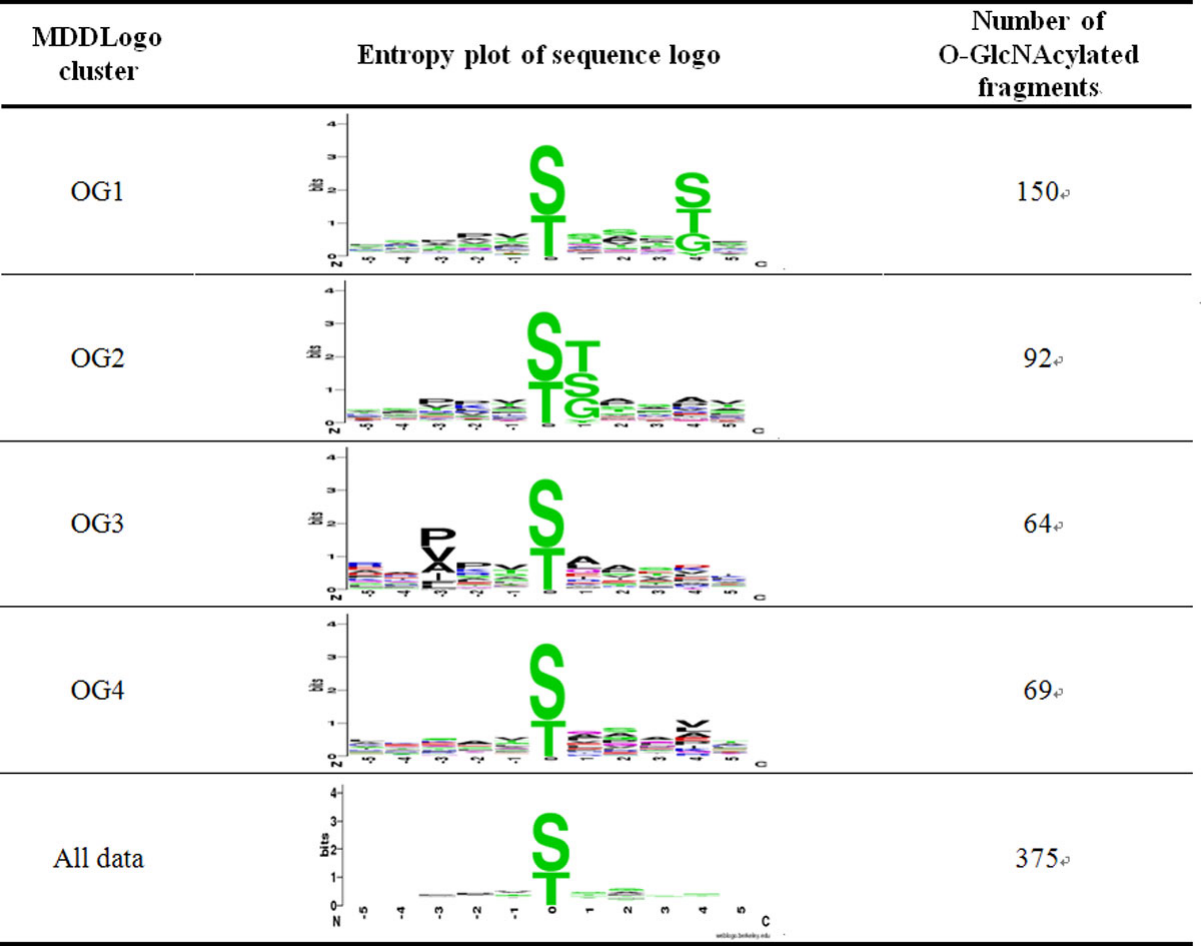
**MDDLogo-identified motifs of O-GlcNAcylation data**.

To further improve the detection of the conserved motifs in large-scale O-GlcNAcylation data set, the MDDLogo was applied to cluster all 375 identified O-GlcNAcylatied peptide sequences, achieving the most significant difference of amino acid composition between positions. With a minimum cluster size of 150 for the O-GlcNAcylated data, we obtained four subgroups shown in Figure 3. The number of positive data in each subgroup was also provided in the last column. With a minimum cluster size >150, no new clusters were obtained, while a minimum cluster size of <150 only generated several similar clusters. Two out of all MDD-clustered subgroups depicted the conserved motifs of polar amino acids (S, T, and G) at position +1 and +4. The third subgroup illustrated the hydrophobic amino acids (P, V, and A) on conserved motifs at specific position -3. However, the fourth subgroup, that contains the remaining 69 O-GlcNAcylation sites, did not have a conserved motif.

### Cross-validation performance

Several related works regarding PTM prediction, KinasePhos [24,25], SNOSite [36], Carboxylator [52], ViralPhos [35], have adopted 20D Binary code Amino, Acid Composition (AAC), Amino Acid Pair Composition (AAPC), Accessible Surface Area (ASA), Position Weight Matrix (PWM), Position-specific scoring matrix (PSSM) to train their predictive models. Here, in order to determine what features had the best performance to classify O-GlcNAcylation from non-O-GlcNAcylation sites, the predictive models were trained with the six features mentioned above. Their predictive power, including sensitivity (Sn), specificity (Sp), accuracy (Acc), and Matthews correlation coefficient (MCC), were then evaluated by using cross-validation. As given in Table 2 The SVM trained with AAC provided predictive sensitivity, specificity, accuracy, and MCC at 0.64, 0.65, 0.65, and 0.17, respectively. As for the model trained with AAPC, the power was 0.66, 0.67, 0.67, and 0.20. Besides, the SVM models trained with ASA or PWM generated the lower discriminating power while the feature of PSSM yielded the best sensitivity of 0.68 and the greatest MCC of 0.22. The specificity and predictive accuracy of the model trained with PSSM was equal to that with binary code and slightly superior to that with other 4 features. Given that PSSM was considered as the best feature for training a model for discrimination of 375 O-GlcNAcylation sites, the predictive sensitivity, specificity, accuracy, and MCC of the best model were 0.68, 0.69, 0.69, and 0.22, respectively. Thus, PSSM was selected as the training feature for the construction of SVM models.

**Table 2:** Five-fold cross validation results on single SVM model trained with various features.

Training features	Number of positive data	Number of negative data	Sn	Sp	Acc	MCC
20D Binary code	375	375	0.66	0.69	0.69	0.21

Amino Acid Composition (AAC)	375	375	0.64	0.65	0.65	0.17

Amino Acid Pair Composition (AAPC)	375	375	0.66	0.67	0.67	0.20

Accessible Surface Area (ASA)	375	375	0.57	0.59	0.59	0.10

Position Weight Matrix (PWM)	375	375	0.62	0.63	0.63	0.14

Position-specific scoring matrix (PSSM)	375	375	0.68	0.69	0.69	0.22

Furthermore, the predictive power for identifying O-GlcNAcylation sites of all four MDD-clustered models was evaluated following five-fold cross-validation. In each SVM model, parameters (Cost and Gamma values) were optimized to achieve high but balanced specificity and sensitivity. The prediction accuracy of all SVM models clustered by MDDLogo is available in Table 3. MDDLogo clusters containing conserved motifs have high predictive accuracies. Subgroup OG1, which contained a conserved S, T, or G residue at position +4, generated an accuracy of 0.81. Subgroup OG2 and OG3, having a conserved S, T, or G residue at position +1, and P, V, or A residue at position -3, both reached the accuracy of 0.79. Besides, the subgroup that did not provide an apparent conserved motif achieved a worse predictive performance. For example, subgroup OG4 had slightly lower accuracy (0.71) than others. Following five-fold cross-validation, MDDLogo-clustered SVMs showed better predictive performance than those lacking MDDLogo. Table 3 shows that the combined MDDLogo-clustered motif SVM model showed higher accuracy with a sensitivity, specificity, accuracy, and a MCC of 0.76, 0.80, 0.78, and 0.37, respectively then the SVM with all O-GlcNAcylation site data which yielded 0.68, 0.69, 0.69, and 0.22, respectively.

**Table 3:** Performance of MDDLogo-clustered SVM models evaluated by five-fold cross validation.

SVM model	Number of positive data	Number of negative data	Sn	Sp	Acc	MCC
All data (Single SVM)	375	375	0.68	0.69	0.69	0.22

Subgroup OG1	150	150	0.80	0.81	0.81	0.41

Subgroup OG2	92	92	0.78	0.79	0.79	0.37

Subgroup OG3	64	64	0.76	0.80	0.79	0.37

Subgroup OG4	69	69	0.70	0.71	0.71	0.25

**Combined performance (MDDLogo-clustered SVMs)**	**375**	**375**	**0.76**	**0.80**	**0.78**	**0.37**

### Independent testing and comparison with other prediction tools

**Figure 4:**
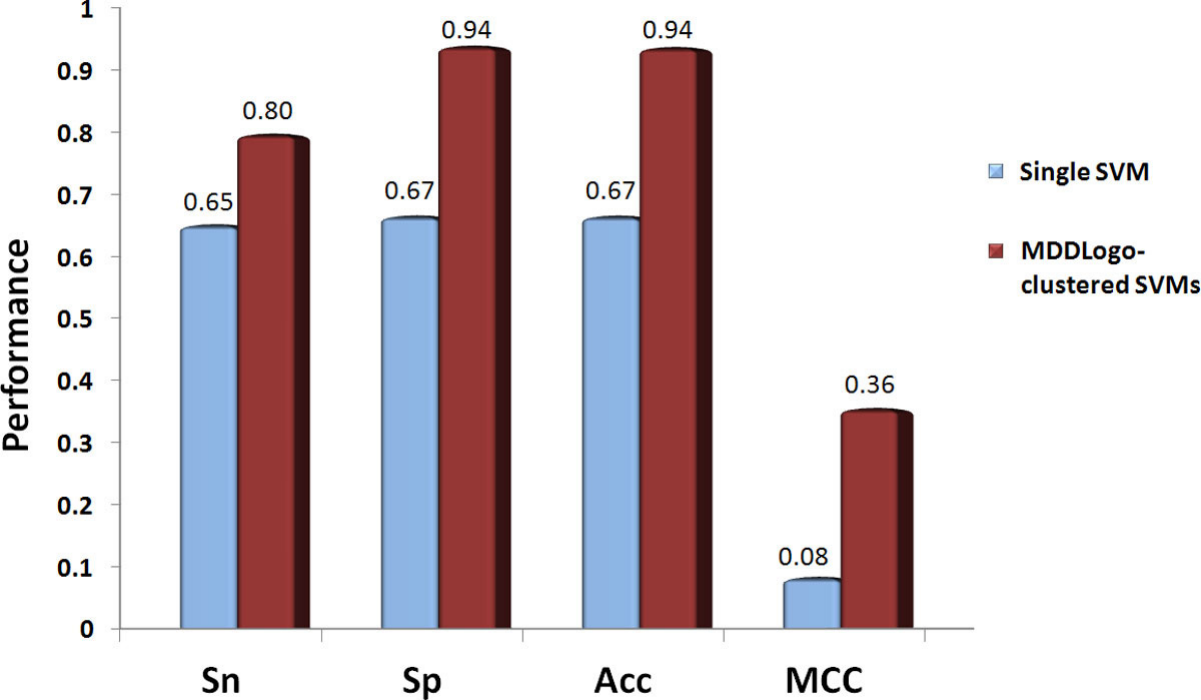
**Comparison of independent testing performance between Single SVM model and MDDLogo-clustered SVM models**.

A non-redundant independent test set consisting of 1048 positive and 64995 negative sites was used to evaluate the MDDLogo-clustered SVMs. The single SVM model achieved a sensitivity of 0.65, a specificity of 0.67, an accuracy of 0.67, and the MCC of 0.08 (shown in Figure 4). Moreover, the integrated SVM models using all the MDDLogo-clustered substrate motifs accomplished a sensitivity of 0.80, a specificity of 0.94, an accuracy of 0.94, and the MCC of 0.36. We concluded that greater prediction power can be obtained by using MDDLogo-clustered SVM models than that by single SVM model. The independent testing demonstrated that the proposed method could provide a promising accuracy for 459 experimentally verified O-GlcNAcylated proteins, which were not considered within the construction of predictive model.

**Figure 5:**
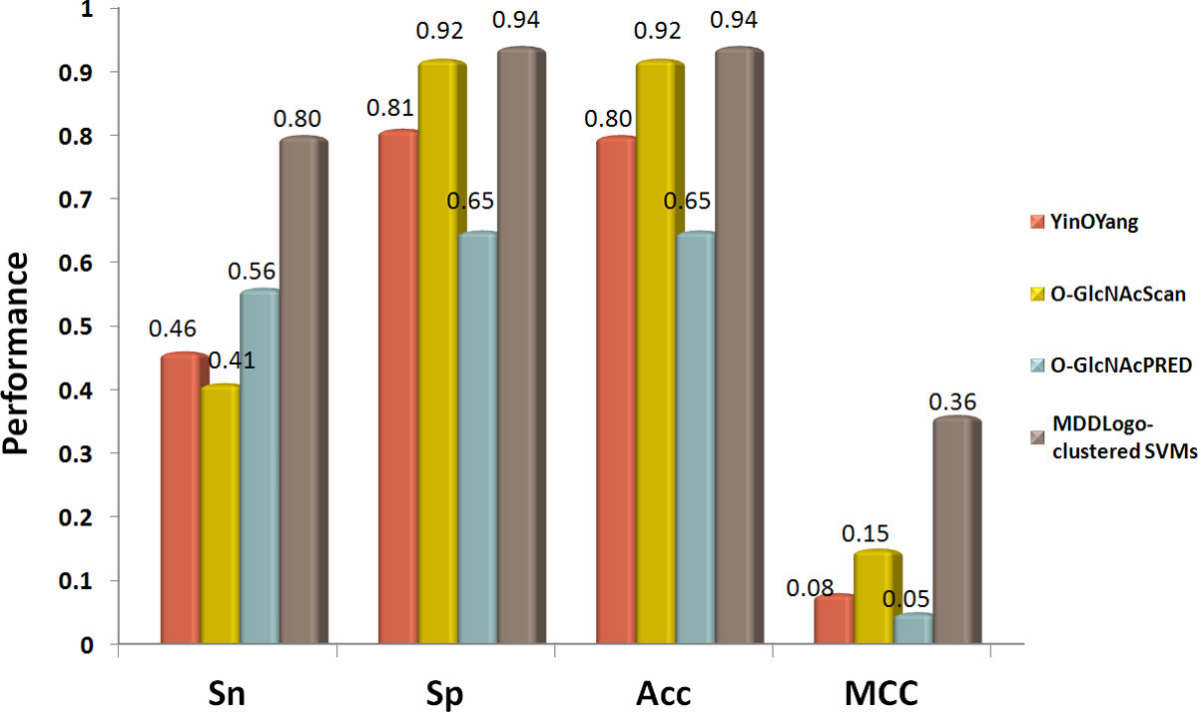
**Comparison of independent testing performance between our method and three available online O-GlcNAcylation site prediction tools**.

**Figure 6:**
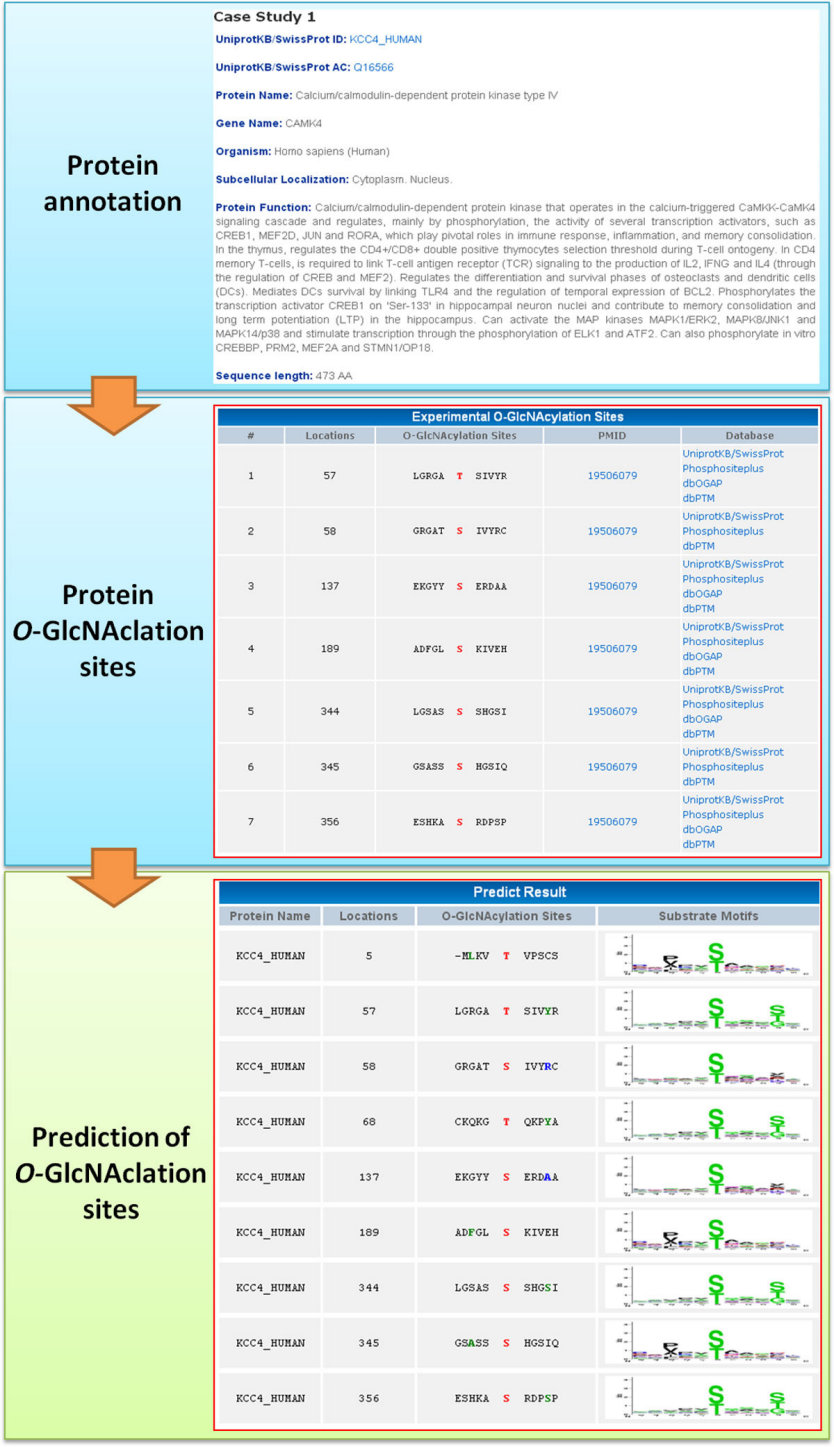
**A case study of O-GlcNAcylation sites prediction on Calcium/calmodulin-dependent protein kinase type IV (CAMK4)**.

To further demonstrate the effectiveness of our method, the independent testing set was used to compare the MDDLogo-clustered SVMs with three popular O-GlcNAcylation site prediction tools, YinOYang [15], O-GlcNAcScan [16], and O-GlcNAcPRED [17]. Figure 5 indicated that the prediction power yielded by our method (0.80 for sensitivity, 0.94 for specificity, 0.94 for accuracy, and 0.36 for MCC) was superior to that by other three prediction tools, especially in sensitivity and MCC, which was almost twice the value of the lowest one. Besides, the proposed method provided comparable specificity and accuracy with that analyzed by O-GlcNAcScan. Overall, the proposed method outperformed the three prediction tools. The detailed independent testing results were presented in Supplementary Table S2 (Additional File 1). Take calcium/calmodulin-dependent protein kinase type IV (CAMK4, Q16566, KCC4_HUMAN) as an example (Figure 6), nine sites including T5, T57, S58, T68, S137, S189, S344, S345, and S356 has been predicted as potential O-GlcNAcylation sites by our model. Among them, T57 S137, S189, S344, and S356 have been confirmed as O-GlcNAcylation sites [55], suggesting the feasibility of this model to sieve out the S/T residues that can be modified by O-GlcNAc moiety.

### Interplay between glycosylation and phosphorylation

Considering the regulatory importance of O-GlcNAcylation of cytosolic and nuclear proteins, there is relatively little understanding of the signature of O-GlcNAcylated proteins and its biological interplay with O-phosphate. To address this issue, we tried to collect the known phosphorylation sites adjacent to the O-GlcNAcylation sites. Within the 375 sequence fragments (starting from upstream -5 to downstream +5) of O-GlcNAcylation sites, there were 207 experimentally verified phosphorylation sites, suggesting the candidate crosstalk between these two modifications. Among the 207 phosphorylation sites, corresponding catalytic kinases of 35 sites has been annotated. Besides, we also found that 80 O-GlcNAcylation sites located nearly with each other (within 10 amino residues). The phosphorylation and O-GlcNAcylation sites that located in the consensus sequence of subgroup OG1 and OG2, with S/T residues at +4 and +1, respectively, were listed in Supplementary Table S3 and S4 (Additional File 1). Take c-myc (MYC_HUMAN) as an example, c-myc has been known to regulate gene transcription in cell proliferation, apoptosis, and metabolism [56]. S62 on c-myc has been known to be a phosphorylation sites while the O-GlcNAcylation of T58 was also reported. Mutagenesis of S62 to Ala showed a marked increase of T58 O-GlcNAcylation. The mediation of O-GlcNAcylation and phosphorylation of T58 and S62 has been demonstrated to regulate the myriad functions of c-Myc in cells [57]. We proposed that the identified substrate motifs in this study may shed light to the study of the site-specific interplay between these two modifications.

## Conclusion

In this study, the substrate motifs of O-GlcNAcylation sites were elucidated by means of identifying the potential substrate specificity of O-GlcNAc transferases. The investigation was done using experimentally verified O-GlcNAcylation sites obtained from dbOGAP. This study explored the use of short linear motifs to further identify O-GlcNAcylated sites. An iteratively statistical method (MDDLogo) was employed to detect substrate motifs on O-GlcNAcylation sites. Based on the MDDLogo-detected substrate motifs, potential O-GlcNAcylation sites were identified according to the corresponding motif signatures. Interestingly, the identified substrate motifs indicated interplay between phosphorylation and O-GlcNAcylation sites. The data may facilitate the study of the cross-talk between these two modifications which can be use to reveal the biological coordination in signaling, transcription, and chronic disease. In the evaluation of predictive power for each single feature, the SVM model trained with PSSM could outperform that trained with other features. Five-fold cross validation further supports our method's ability to identify O-GlcNAcylation sites containing the MDDLogo-identified substrate motifs. Furthermore, an independent test done by using data not included in the model training confirmed the ability of MDDLogo-clustered SVMs.

In addition to the consideration of linear sequence motifs, structural recruitment is very important in the investigation of O-GlcNAcylated substrate specificity. However, with limited information regarding O-GlcNAcylated sites on protein three-dimensional (3D) structures, the structural environment of O-GlcNAcylation sites could not be investigated with sufficient experimental data [58]. This was the main reason why this work developed a method to characterize the potential substrate motifs for O-GlcNAcylation sites. The approach offered the clues regarding the specificity of site information of O-GlcNAcylation. It would be noticed, however, that the further acquisition of experimentally verified O-GlcNAcylation sites is required to identify more meaningful substrate motifs. Also, a more abundant set of experimentally verified O-GlcNAcylation sites on protein 3D structures could be used to study the substrate recruitment of O-GlcNAc transferase. These developments could benefit from our method by obtaining a more accurate identification of O-GlcNAcylation sites.

## Competing interests

The authors declare that they have no competing interests.

## Authors' contributions

TYL and YJC conceived and supervised the project. CTL and HJK carried out the design, computational analyses, implemented the web-based tool, and write the manuscript, with inputs from HYW, YJC, and TYL. All authors read and approved the final manuscript.

## Supplementary Material

Additional file 1 **Supplementary Tables and Figures**. Contains additional Tables and Figures showing further results in this study Click here for file

## Declarations

Publication charge for this work was funded by MOST grant 100-2628-M-001-003-MY4 to YJC.

This article has been published as part of *BMC Bioinformatics *Volume 15 Supplement 16, 2014: Thirteenth International Conference on Bioinformatics (InCoB2014): Bioinformatics. The full contents of the supplement are available online at http://www.biomedcentral.com/bmcbioinformatics/supplements/15/S16.
