# Supplementary material for: Characterization and identification of protein O-GlcNAcylation sites with substrate specificity
Source: BMC Bioinformatics. 2014 Dec 8;15(Suppl 16):S1. doi: 10.1186/1471-2105-15-S16-S1 (PMC4290634; doi:10.1186/1471-2105-15-S16-S1)
Supplement: Additional file 1 — Supplementary Tables and Figures. Contains additional Tables and Figures showing further results in this study [file 1471-2105-15-S16-S1-S1.pdf]

Supplementary Figures S1-S2 and Tables S1-S4

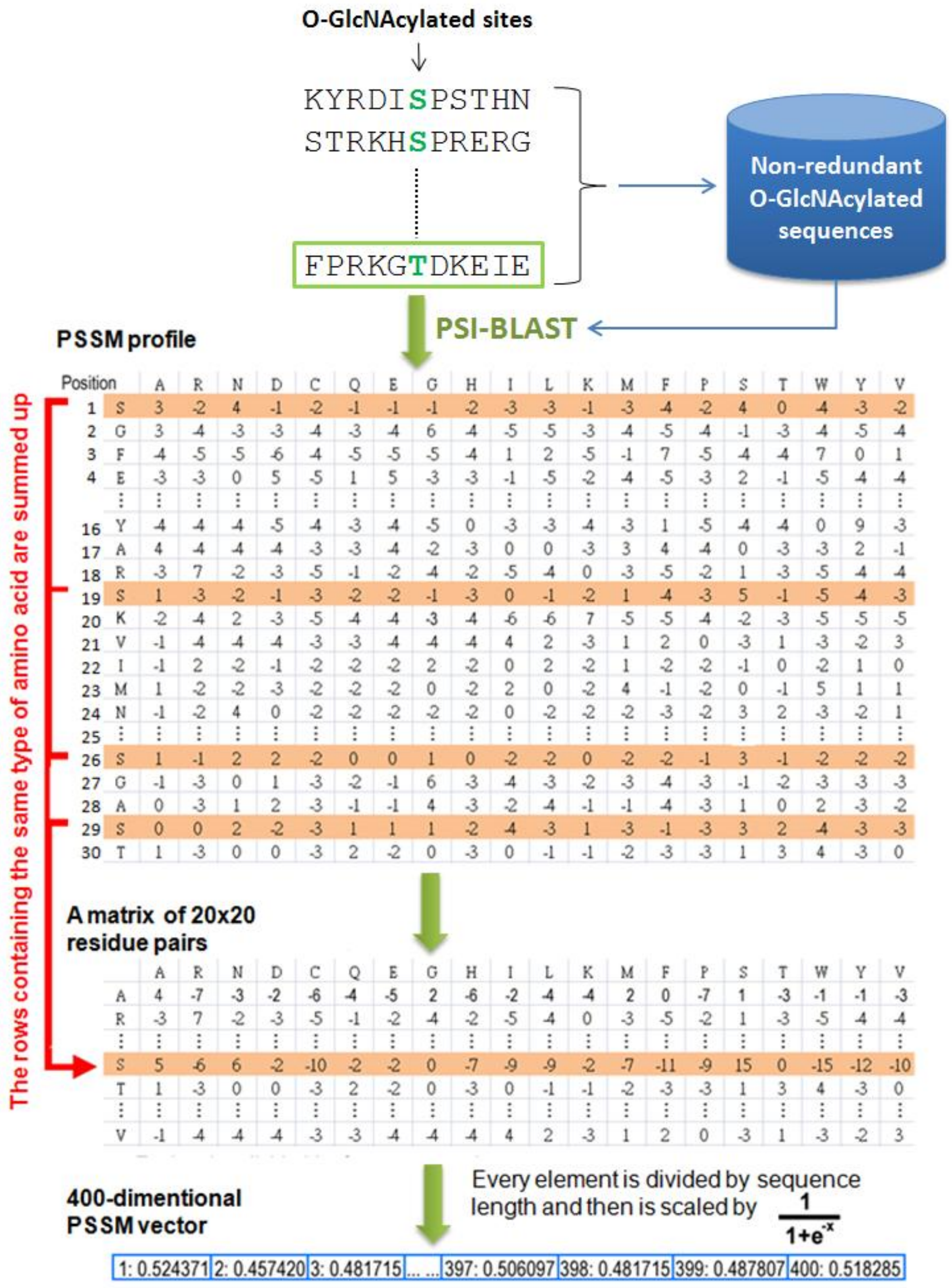

Figure S1. The encoding scheme of position-specific scoring matrix (PSSM) with 400-dimensional vector.

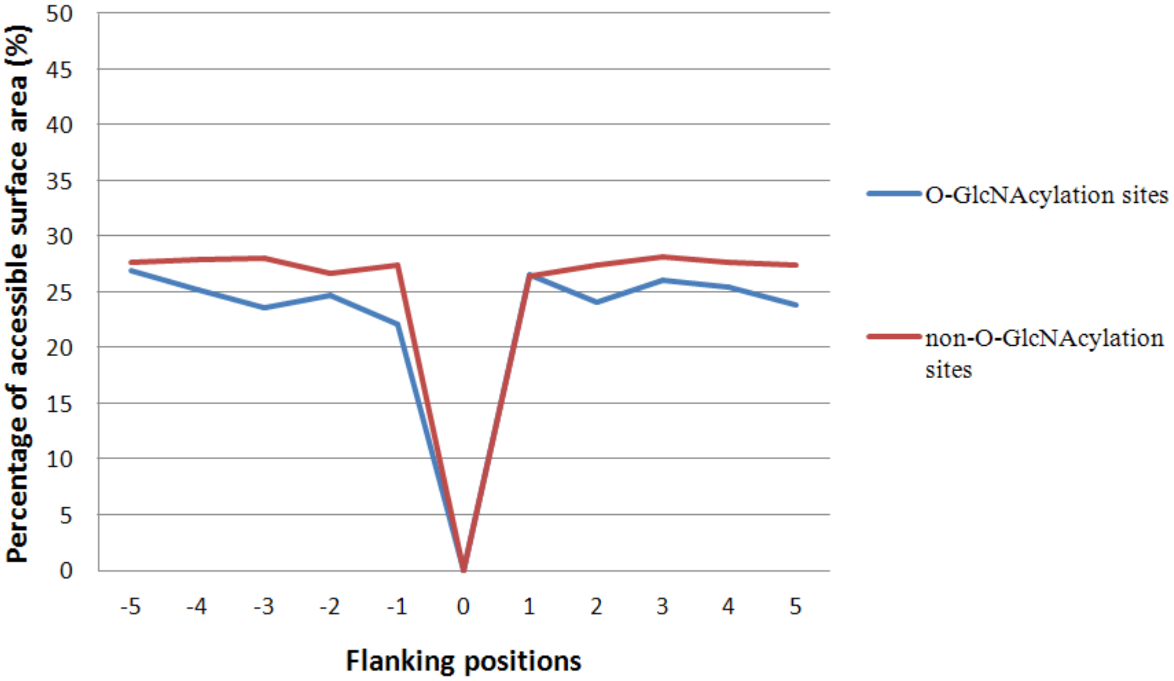

Figure S2. Comparison of solvent-accessible surface area between O-GlcNAcylated and non-O-GlcNAcylated sites.

Table S1. The amino acids group of MDDLogo used in this study.

Chemical properties	Amino acids
**Polar group**	Glycine (G), Serine (S), Threonine (T), Cysteine (C), Glutamine (Q), Asparagine (N)
**Acidic group**	Aspartic acid (D), Glutamic acid (E)
**Basic group**	Lysine (K), Arginine (R), Histidine (H)
**Hydrophobic group**	Alanine (A), Valine (V), Leucine (L), Isoleucine (I), Proline (P), Methionine (M)
**Aromatic group**	Phenylalanine (F), Tyrosine (Y), Tryptophan (W)

Table S2. The detailed independent testing results between our method and other three O-GlcNAcylation prediction tools.

Predictive tools	TP	FP	TN	FN	Sn	Sp	Acc	MCC
Single SVM	683	21713	43282	365	0.65	0.67	0.67	0.08
Two-layered SVMs	836	3934	61061	212	0.80	0.94	0.94	0.36
YinOYang	482	12349	52646	566	0.46	0.81	0.80	0.08
O-GlcNAcScan	430	5199	59796	618	0.41	0.92	0.92	0.15
O-GlcNAcPRED	587	22748	42247	461	0.56	0.65	0.65	0.05

Table S3. The phosphorylation and O-GlcNAcylation sites that located in the consensus sequence of subgroup OG1 with S/T residues at +4.

Entropy plot of OG1 sequence logo

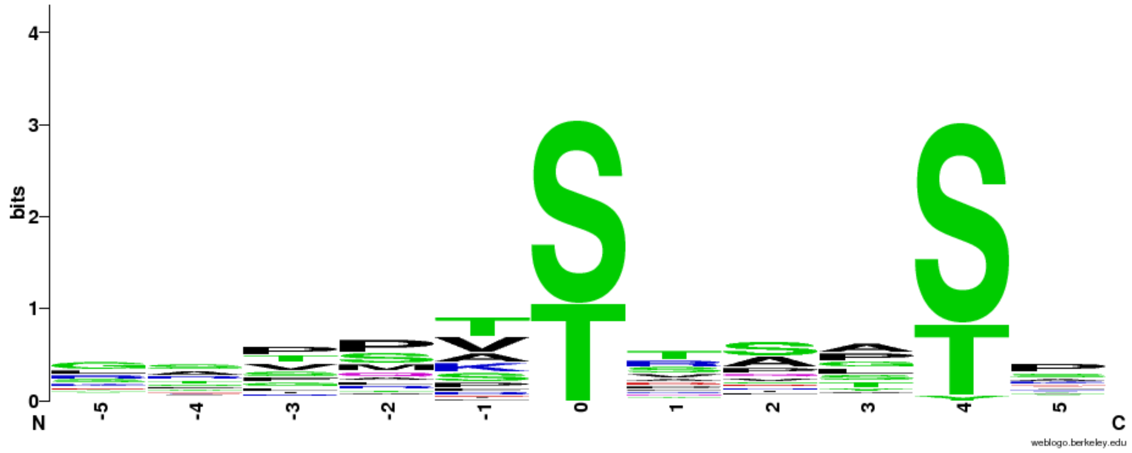

Gene Symbol	O-GlcNAc site	Consensus Suence[a]	PubMed ID[b]	Modification on +4 position[c]
ABLM3_MOUSE	419	LDVRS S TPTSY	19458039	O-GlcNAc
AKT1_HUMAN	308	GATMK T FCGTP	22629392<br>22322096	O-GlcNAc<br>Phosphothreonine
AKT1_HUMAN	473	HFPQF S YSASG	22322096	Phosphoserine
BPTF_HUMAN	1749	ENCAK S TVTTT	20068230	O-GlcNAc
BPTF_HUMAN	2094	KMVLT T KVGSP	18669648<br>19690332<br>20068231<br>21406692<br>19413330	Phosphoserine
BSN_MOUSE	1395	GSPAT T ASPTR	16452088	O-GlcNAc
BSN_MOUSE	1407	YMTPT S PAGSE	16452088<br>22645316	O-GlcNAc
BSN_MOUSE	2054	DLRHP T DLLSH	16452088<br>19458039<br>22645316	O-GlcNAc
BSN_MOUSE	2070	RYSSV S NIYSD	22645316<br>18034455	O-GlcNAc<br>Phosphoserine
BSN_MOUSE	2941	LAGPT T VPATK	16452088<br>19458039<br>22645316	O-GlcNAc
CEBPB_MOUSE	180	GSLST S SSSSP	15985551	Phosphoserine

					17110376<br>17264204<br>17601773<br>19478079<br>21183079<br>21659604<br>21659605<br>22262865<br>15985552	(GSK-3_beta)
CRTC2_HUMAN	173	RTSSD	S	ALHTS	19690332<br>18669648	Phosphothreonine
CTND2_MOUSE	453	GTFRT	S	TAPSS	16452088<br>16452087	O-GlcNAc<br>Phosphoserine
DEMA_MOUSE	285	LHSGT	S	KSSSL	16452087<br>17114649<br>18388127<br>19737024<br>20415495<br>21183079	Phosphoserine
HCFC1_HUMAN	579	TTIVK	T	MAVTP	20068231	Phosphothreonine
K1C18_HUMAN	30	GARPV	S	SAASV	16083285<br>9524113<br>10623656<br>14694114<br>18669648<br>19415658<br>20044836<br>20068231<br>15368451<br>19007248	Phosphoserine<br>(CDK1)
K1C18_HUMAN	49	SRISV	S	RSTSF	10623656<br>18669648<br>20044836<br>20068231<br>17081983<br>7523419<br>1374067<br>17924679	Phosphoserine<br>(PKC_epsilon;CAMK2A;PRKCA;PRKCE;RPS6KA3;AURKA)

					19007248<br>15368451<br>16424149	
KCC4_HUMAN	356	ESHKA	S	RDPSP	17192257<br>19690332<br>20363803<br>19413330	Phosphoserine (CAMK4)
LMNA_HUMAN	612	VGGPI	S	SGSSA	17081983<br>18669648<br>19691289<br>20068231	Phosphoserine
MYC_HUMAN	58	FELLP	T	PPLSP	10551811<br>10866684<br>11018017<br>11243879<br>11904304<br>14563837<br>15103331<br>15150404<br>15175348<br>15210690<br>16452238<br>16483932<br>1651323<br>16537924<br>16855632<br>17157259<br>17634226<br>18220336<br>18408012<br>18669648<br>18701488<br>19028839<br>19131971<br>20068231<br>20140016<br>21406692<br>21659604	Phosphoserine (MAPK1;GSK3;CDK2)

					21808024<br>22025562<br>22115753<br>8386367<br>17081983<br>17558397<br>19966300<br>20713526	
MYH6_RAT	622	MATLF	S	TYASA	18988896	O-GlcNAc
NCOR1_HUMAN	1487	RRTPV	S	YQNTM	17081983	Phosphothreonine
NFL_MOUSE	48	YSAPV	S	SSLSV	16452088	O-GlcNAc
NFM_MOUSE	46	SRGSP	S	TVSSS	16452088	O-GlcNAc
NFM_MOUSE	47	RGSPS	T	VSSSY	16452088	O-GlcNAc
P121A_HUMAN	693	TKPQA	T	SAPSP	18669648<br>19413330	Phosphoserine
RRP1B_HUMAN	731	LKTPT	S	SPASS	17081983<br>18669648<br>19664995<br>19651622<br>19691289<br>20068231<br>19690332<br>21659604<br>22115753	Phosphoserine
SHAN2_MOUSE	1292	PEVMS	T	VSGTR	16452088	O-GlcNAc
SKT_MOUSE	357	SSLPV	S	RSISP	19367708	Phosphoserine
SP1_RAT	699	KRFMR	S	DHLSK	16332679<br>15561949<br>12900380<br>9111324<br>16365142<br>16052526	O-GlcNAc<br>Phosphoserine
SPTB2_HUMAN	2324	STPAS	S	RAQTL	17081983<br>18669648<br>19651622<br>19362540	Phosphothreonine
SRBS1_MOUSE	1201	LPYSS	S	PSRSA	20139300<br>21183079	Phosphoserine

SRF_HUMAN	309	APVSA	S	VSPSA	1512232<br>12684542	O-GlcNAc
SYN1_MOUSE	564	GAPQA	T	RQASI	16452087<br>19737024<br>20415495	Phosphoserine
SYN1_RAT	562	GPPQA	T	RQASI	3118371	Phosphoserine<br>(CaMK2)
TAU_RAT	711	KSPVV	S	GDTSP	15249677<br>15375165<br>15719129<br>15992372<br>16397900<br>16452087<br>16675464<br>17085446<br>17537547<br>18245083<br>18587388<br>19054758<br>19451179<br>20308788<br>21489990<br>8245007	Phosphoserine<br>(CK1;PDPK1)
VIME_HUMAN	34	SYVTT	S	TRTYS	18083107<br>20090780<br>21253578	Phosphotyrosine
YTHD3_MOUSE	201	IGGDL	T	AAVTK	22645316	O-GlcNAc
ZC3HE_HUMAN	369	ESVTK	T	TNYST	19413330	Phosphoserine

[a]Residues colored by blue is the annotated O-GlcNAylation sites in the training data.

[b]PubMed ID that reported the modification on +4 position to the central residue

[c]Reported phosphorylation or O-GlcNAcylation on +4 position

Table S4. The phosphorylation and O-GlcNAcylation sites that located in the consensus sequence of subgroup OG2 with S/T residues at +1.

Entropy plot of OG2 sequence logo

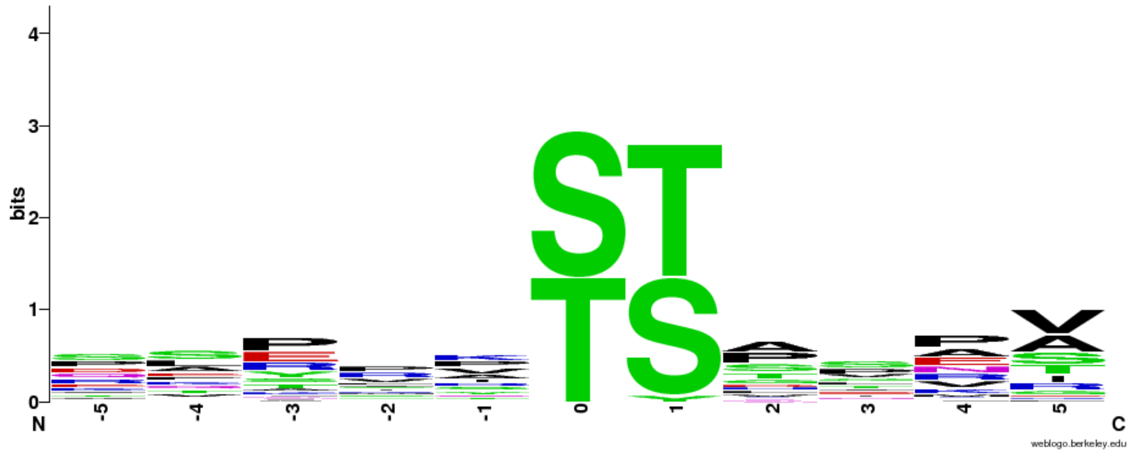

Gene Symbol	O-GlcNAc site	Consensus Suence	PubMed ID	Modification on +1 position
BSN_MOUSE	1418	RSPST S STIHS	16452088	O-GlcNAc
BSN_MOUSE	2067	PLRRY S SVSNI	16452087<br>20415495<br>21183079<br>6452088<br>19458039	O-GlcNAc<br>Phosphoserine
BSN_MOUSE	2188	RQLLP S TATVR	16452088<br>22645316<br>19458039	O-GlcNAc
BSN_MOUSE	2317	EEPFS T TAPAV	16452088<br>19458039<br>22645316	O-GlcNAc
DLGP1_RAT	525	SSPPR T TTTVR	19458039	O-GlcNAc
EMSY_HUMAN	556	GGKII S SNIVS	20068230	O-GlcNAc
EMSY_MOUSE	499	TYVKT T SGSII	21606357	O-GlcNAc
FOXO1_HUMAN	317	TFRPR T SSNAS	18420577	O-GlcNAc
H31_HUMAN	11	QTARK S TGGKA	18066052<br>18243098<br>19407812<br>20948546<br>21357687<br>21514157<br>12560483	Phosphothreonine (DAPK3;PKC;CHEK1)

HCFC1_HUMAN	800	PITII T TKVMT	20068230<br>23335398	O-GlcNAc
K2C8_HUMAN	13	KSYKV S TSGPR	19007248	Phosphothreonine
KPCG_RAT	689	DARSP T SPVPV	18295358	O-GlcNAc
LT_SV40	111	SEEMP S SDDEA	9499386	O-GlcNAc<br>Phosphoserine
MYH6_RAT	1470	QSELE S SQKEA	18988896	O-GlcNAc
NU153_HUMAN	1112	QQEPV T STSLV	20068230	O-GlcNAc
NUP98_HUMAN	567	PKALQ T TGTAK	20068230	O-GlcNAc
P53_HUMAN	149	QLWVD S TPPPG	11285227<br>12628923<br>17906639<br>21127074	Phosphothreonine
PCLO_MOUSE	2686	DVKRQ T TANEV	22645316	O-GlcNAc
PCLO_MOUSE	2889	AQQPA T TLPED	16452088<br>19458039<br>119458039<br>15648052<br>22645316	O-GlcNAc
PCLO_MOUSE	2993	STPQY S TARMT	16452088	O-GlcNAc
PHB_HUMAN	258	RSRNI T YLPAG	19238206<br>22410782	Phosphotyrosine
RBP2_HUMAN	1399	VFTPK T SPENV	20068230<br>18669648<br>19651622<br>20068231	O-GlcNAc<br>Phosphoserine
RPRD2_HUMAN	399	EKSAV S TSVPT	20068230<br>19413330	O-GlcNAc<br>Phosphothreonine
SON_HUMAN	252	AAPVP T TTLVL	20068230	O-GlcNAc
SP1_RAT	641	KVYGK T SHLRA	16332679<br>15561949<br>12900380<br>9111324<br>16365142<br>16052526	O-GlcNAc<br>Phosphoserine
SRBS1_MOUSE	1200	DLPYS S SPSRS	16452088<br>15648052	O-GlcNAc<br>Phosphoserine

			18056256<br>19367708<br>19737024<br>21183079<br>21659604	
SYN1_MOUSE	55	SAERA S TAAPV	16452088<br>19004831<br>16452087	GlcNAc<br>Phosphothreonine
TPR_HUMAN	1676	PTPVV S TPSKV	18669648<br>19413330<br>17446864<br>18847512	Phosphothreonine
UBP2L_HUMAN	782	RSSVA T TSGKA	20068230	O-GlcNAc
ZFR_HUMAN	148	YSYVR S TAPAV	20068230	O-GlcNAc
